# Structural equation model analysis of the effect of visceral fat on osteoporosis

**DOI:** 10.1186/s13018-024-04888-5

**Published:** 2024-07-16

**Authors:** Min Tong, Yuanyuan Li, Kai Rong, Qi Sun, Jianghong Dai, Yifei Huang

**Affiliations:** 1 The Second Spine Department, Traditional Chinese Medicine Affiliated Hospital of Xinjiang Medical University, No. 116 Huanghe Road, Urumqi, 830000 China; 2 School of Public Health, Xinjiang Medical University, Urumqi, 830011 China; 3 The Fourth Orthopedic Department, Traditional Chinese Medicine Affiliated Hospital of Xinjiang Medical University, Urumqi, 830000 China; 4 Medical Research Design and Data Analysis Center, Traditional Chinese Medicine Affiliated Hospital of Xinjiang Medical University, Urumqi, 830000 China

**Keywords:** Osteoporosis, Structural equation model, Visceral fat

## Abstract

**Background:**

Osteoporosis is a considerable public health challenge in Moyu County, Xinjiang. Here, we evaluated the influencing factors of osteoporosis in this region.

**Methods:**

We recruited 7,761 participants and randomized them into normal and osteoporotic populations based on T-score. The effects of general conditions, body composition, calcium sources and exercise, respiratory exposure, and daily diet on osteoporosis were analyzed. Furthermore, a structural equation model was constructed to uncover the direct and indirect influencing factors of osteoporosis.

**Results:**

Among the participants, 1,803 (23.23%) had normal bone mass while 1,496 (19.28%) had osteoporosis. The univariate analysis showed significant differences in the general conditions, body composition, calcium sources and exercise, respiratory exposure, and daily diet. Stratification based on age (45 years) and body mass index (BMI) (18.5 kg/m^2^) showed variations in the body composition between the two groups; however, the visceral fat differed significantly. Logistic regression analysis affirmed the association of visceral fat index as it was included in all equations, except for age and female menopause. The structural equation exhibited that the general conditions, body composition, and, calcium sources, and exercise were direct factors of osteoporosis, while respiratory exposure and daily diet were indirect factors. The standardized path coefficient was highest in general conditions, followed by body composition, and lastly, calcium sources and exercise.

**Conclusion:**

Obesity, besides age and female menopause, is also an influencing factor of osteoporosis. The visceral fat index plays a vital role in osteoporosis. Our findings may provide experimental evidence for early prevention and treatment of osteoporosis.

**Supplementary Information:**

The online version contains supplementary material available at 10.1186/s13018-024-04888-5.

## Background

Osteoporosis is a prevalent bone disease that is characterized by low bone mass, deterioration of bone tissue, and destruction of bone structure [1]. The etiology and influencing factors of osteoporosis have been the subject of extensive research [2]. The factors known to affect this disease include age [3], female menopause smoking, drinking, dietary supplements [4], (such as calcium and vitamin D), exercise, and obesity [5].

The relationship between obesity and osteoporosis is complex, and no clear conclusion has been reached yet. On the one hand, obesity may act as a protective factor against osteoporosis and fractures. One previous study [6] has indicated a direct relationship between body mass index (BMI) and bone mineral density (BMD). A subsequent study [7] in postmenopausal women has linked higher BMI to a lesser likelihood of hip fracture. On the other hand, several studies, including meta-analyses, have reported various fractures in women and elderly men after adjusting for BMD [8, 9]. Obesity can be both a risk and a protective factor, and a high BMI is also considered a risk factor for osteoporosis. Research [10] has shown that women who are not obese after menopause tend to have lower bone density and higher rates of osteoporosis. Additionally, obesity may increase the risk of osteoporosis, as adipose tissue can release hormones and cytokines that promote the development of osteoporosis .

The uncertainty regarding the relationship between obesity and osteoporosis may stem from the fact that previous studies used BMI as the primary measure of obesity. Recent studies [11, 12] that focused on the relationship between body composition and health, particularly cardiovascular health, have resolved many of the paradoxes related to obesity and identified excessive body fat as a risk factor. Body composition is composed mostly of fat and non-fat mass, and the fat includes visceral fat and subcutaneous fat. Evidence has shown that non-fat mass is positively correlated with bone density in all skeletal sites and is a protective factor for osteoporosis [13]. Nevertheless, there is an ongoing debate about the relationship between fat mass, different BMI values and standards, and osteoporosis [14, 15]. Furthermore, limited research has focused on visceral fat, which is closely linked to obesity [16].

The structural equation model provides a clear analysis of how individual indicators affect the overall situation and their relationship with each other. In comparison to traditional multivariate regression analysis, it can simultaneously consider the relationships between multiple variables, providing a more comprehensive and accurate analysis for identifying influencing factors. Previous studies [17, 18] have analyzed the influencing factors of osteoporosis in elderly and postmenopausal women using the structural equation model. However, studies on the effects of obesity, abdominal obesity, and visceral fat on osteoporosis are limited. Furthermore, the effect of visceral fat on young adults and male youth has been largely unreported.

Herein, this article investigated the direct and indirect influencing factors of osteoporosis in the rural residents of Moyu, Xinjiang. Data were collected by detailed questionnaires and dietary surveys. The body composition was tested. The structural equation model was constructed to evaluate the influencing factors of osteoporosis. The findings may provide a theoretical basis for the prevention and treatment of osteoporosis.

## Methods

### Sample size calculation

The villages were selected according to the principle of regional representativeness and operability. A multi-stage random cluster sampling method was used to determine the appropriate sample size, estimating a disease prevalence of 15% with a relative error rate of 5‰. The sample size was calculated with the formula $\text{n}=\frac{{{\text{u}}_{{\upalpha }}}^{2}\text{p}\left(1-\text{p}\right)}{{{\updelta }}^{2}}$. Finally, we calculated a sample size of 7,056 individuals.

### Study participants

We enrolled participants from the Xinjiang Multi-Ethnic Cohort [19]. In detail, between May and November of 2018, a survey of 102 natural villages in seven townships covering 75% of the area in Moyu County, Hotan Prefecture, Xinjiang was conducted. A total of 7,872 individuals were recruited, 7,761 of whom received both bone density and body composition tests. Inclusion criteria: (1) Age between 20 and 75 years old; (2) Individuals willing to participate in the questionnaire survey and physical measurement. Exclusion Criteria: (1) Individuals who cannot cooperate with bone density testing; (2) Pregnant women; (3) Individuals with severe internal or surgical diseases. All participants provided written informed consent. This study was conducted according to the Declaration of Helsinki and approved by the Ethics Committee of Xinjiang Uygur Autonomous Region Traditional Chinese Medicine Hospital (approval no. 2018XE0108).

### Questionnaire survey

The baseline data of participants were collected using the unified questionnaire from the “Northwest Region Natural Population Cohort Construction” project. The collected data included demographic characteristics, lifestyle factors (such as smoking, drinking, tea drinking, and physical activity), disease history (health status, history of chronic diseases, and female reproductive history), dietary conditions, etc.

### Body composition

The body composition was measured using the bioelectrical impedance analyzer (TANITA DC-430MA; TANITA Corporation, Tokyo, Japan). The readings for height and weight were accurate at 0.1 cm and 0.1 kg, respectively. Waist circumference was measured from the midpoint between the upper edges of the hips to the lower edge of the ribcage during quiet exhalation, with a reading accuracy of 0.1 cm. The test results included body weight, body fat percentage, non-fat mass, muscle mass fraction, muscle mass, obesity level, visceral fat index (VFI), body water percentage, and basal metabolism.

### BMD T-score measurement and osteoporosis diagnosis

BMD T-score was measured at the left calcaneus using SONOST-2000 (OsteoSys, Korea) [20, 21]. This equipment was linked to a computer with analysis software based on Asian standards. The broadband ultrasound attenuation (decibels/MHz) and speed of sound (m/s) were measured. The stiffness index was calculated using the formula: stiffness index = 0.67 × (broadband ultrasound attenuation) + 0.28 × (speed of sound) − 420. The measurement results were compared to the Asian standards equipped on the computer to generate a BMD T-score for further evaluation. The BMD T-score was determined as the tested value = (estimated value – average value for individuals of the same gender) / standard deviation. According to the diagnosis standard of the World Health Organization, a BMD T-score ≥ − 1.0 was considered normal, -1.0 > BMD T-score >-2.5 was considered osteopenia, and a BMD T-score ≤-2.5 was considered osteoporosis.

### Statistical analysis

Quantitative data of normal distribution are described by mean and standard deviation and were tested using an F-test followed by LSD. Qualitative data, which are described using absolute numbers and composition ratios, were analyzed using a chi-square test.

The stratified analysis was conducted based on the Chinese adult BMI classification standard. A BMI greater than or equal to 18.5 Kg/m^2^ was categorized as underweight, 18.5 Kg/m^2^< BMI<24.0 Kg/m^2^ was considered normal, 24.0 Kg/m^2^< BMI<28.0 Kg/m^2^ was classified as overweight, and BMI ≥ 28.0 Kg/m^2^ was defined as obese. The classification for young adults was set at 45 years of age, considering factors such as the World Health Organization’s classification for young adults and menopausal status in females.

A multivariate logistic regression analysis was conducted to analyze the factors affecting osteoporosis, using the normal group as the control and osteoporosis as the event. The influencing factors were stratified according to the entire population, people with BMI > 18.5 Kg/m^2^, males with BMI > 18.5 Kg/m^2^, females with BMI > 18.5 Kg/m^2^, males under the age of 45 with BMI > 18.5 Kg/m^2^, and females under the age of 45 with BMI > 18.5 Kg/m^2^. A two-sided *P* value less than 0.05 was considered significant. R4.1.1 was used for statistical analysis and graphical plotting.

### Structural equation modeling

The structural equation model was constructed using SPSS AMOS 26.0, according to the following three steps. (1) Theoretical model establishment: This step involved proposing theoretical models based on literature review, data collection, and work experience. (2) Exploratory factor analysis: The collected data variables underwent factor analysis, wherein variables that were not reasonably classified were removed to reduce dimensionality and achieve maximum cumulated explained variance. The final variables were then selected. (3) Confirmatory factor analysis: This step involved identifying the model, sample size, parameter estimation, and fitting parameters, and standardizing model path coefficients and factor loads. Model modification followed standardization. The measurement reliability of the model was assessed by the degree to which measured indicators could accurately reflect the connotation of latent variables, measured by the squared multiple correlation coefficient (R^2^) of the measured variables. Generally, R^2^ > 0.5 was considered reliable.

## Results

### Baseline data and univariate analysis

**Fig. 1:**
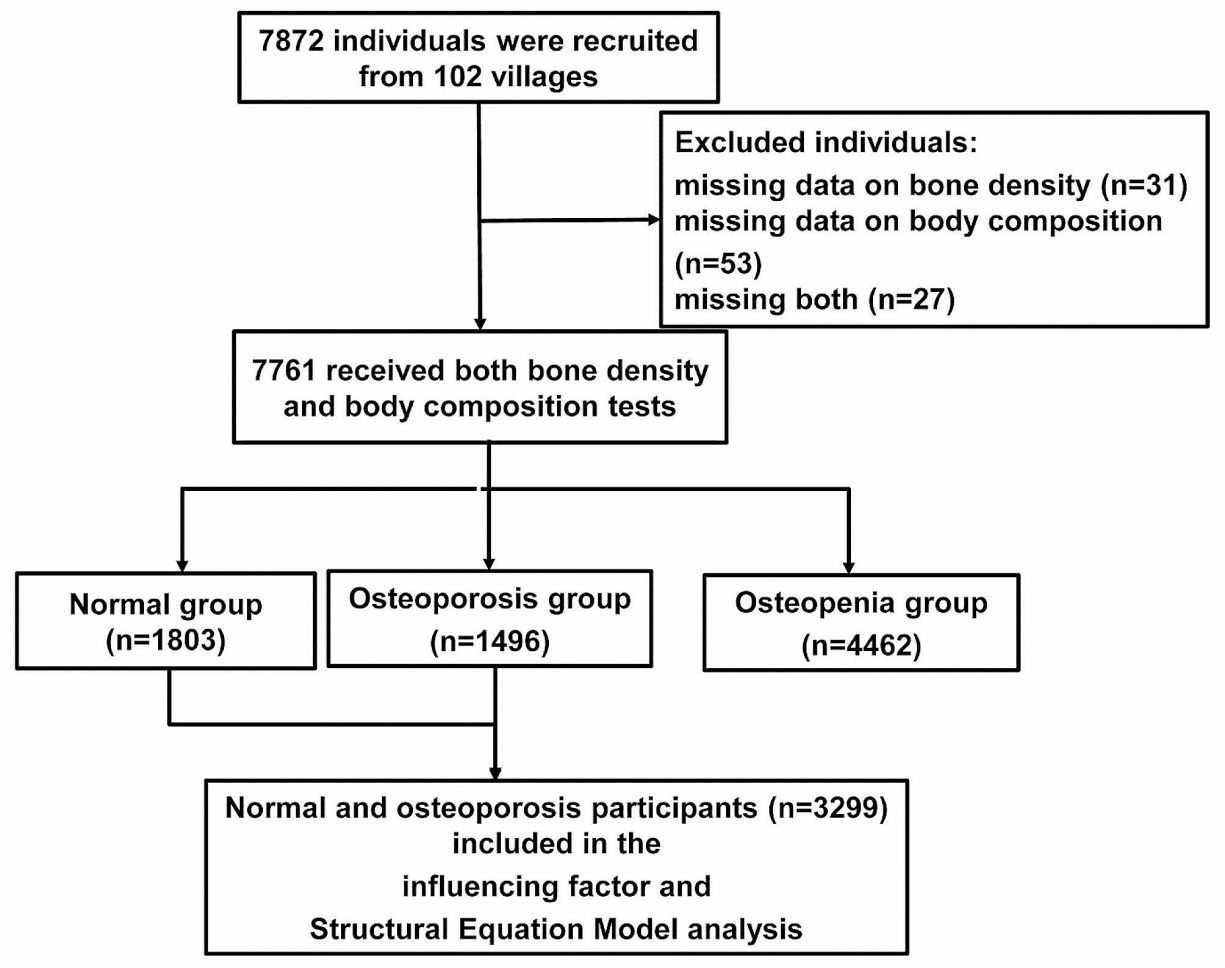
The participant selection and the number of participants during different statistical analyses

In this study, 7761 participants were surveyed and Fig. 1 shows the participant selection and the number of participants during different statistical analyses. The participants were classified into three groups based on the BMD T-score: normal (23.23%; 1,803), osteoporosis (19.28%; 1,496), and osteopenia (57.49%; 4,462). Univariate analysis revealed that age, height, gender, menopause, occupation, education level, previous diseases, rheumatoid arthritis, fractures, calcium sources and exercise (calcium or vitamin D supplements, Labor, and dairy product intake), daily diet (carbonated drinks, meat, and tea consumption), and respiratory exposure (heating or cooking fuel, secondhand smoke exposure, and smoking) showed statistically significant differences among the three groups (*P* < 0.05), as presented in Table 1.

**Table 1:** Univariate analysis of osteoporosis and osteopenia (*n* = 7761)

Variables		Normal (*n* = 1803)	Osteoporosis (*n* = 1496)	Osteopenia (*n* = 4462)	t/χ^2^	*P*
General condition						
Age		49.47 ± 9.47	57.7 ± 9.66^*#^	52.99 ± 9.87	292.48	< 0.001
Height (cm)		158.19 ± 7.88	155.19 ± 9.09^*#^	156.69 ± 8.51	9.591	< 0.001
Sex	Male	734(40.71)	525(35.09) ^*^	1682(37.7)	11.134	0.004
	Female	1069(59.29)	971(64.91) ^*^	2780(62.3)		
Menopause	No	774(42.93)	236(15.78) ^*#^	1339(30.01)	482.756	< 0.001
	Yes	295(16.36)	735(49.13)	1441(32.29)		
	Not applicable	734(40.71)	525(35.09)	1682(37.7)		
Occupation	Farmer	1725(99.02)	1447(99.18)	4288(99.21)	0.54	0.7633
	Others	17(0.98)	12(0.82)	34(0.79)		
Education level	No schooling	19(1.08)	26(1.8) ^*#^	51(1.18)	64.783	< 0.001
	Elementary school	1482(84.59)	1099(76.11)	3483(80.29)		
	Junior high School	134(7.65)	100(6.93)	321(7.4)		
	High school/vocational school	117(6.68)	219(15.17)	483(11.13)		
Previous illness	No	1513(85.87)	995(67.83) ^*#^	3356(76.99)	148.913	< 0.001
	Yes	249(14.13)	472(32.17)	1003(23.01)		
Rheumatic arthritis	No	1551(86.02)	1219(81.48) ^*^	3745(83.93)	12.502	0.002
	Yes	252(13.98)	277(18.52)	717(16.07)		
Fracture	No	1711(94.90)	1378(92.11) ^*^	4174(93.55)	10.61	< 0.001
	Yes	92(5.10)	118(7.89)	288(6.45)		
Calcium sources and exercise						
Calcium or vitamin D supplements	Both are absent	501(29.98)	303(21.6) ^*#^	1113(26.83)	34.48	< 0.001
	Only one supplement	781(46.74)	785(55.95)	2060(49.66)		
	Both are present	389(23.28)	315(22.45)	975(23.51)		
Labor	Rarely	271(15.35)	314(21.42) ^*#^	656(15.02)	40.33	< 0.001
	3–5 times a week	630(35.69)	537(36.63)	1676(38.38)		
	Every day	864(48.95)	615(41.95)	2035(46.6)		
Dairy product intake	Extremely rare	31(1.76)	24(1.65) ^*^	99(2.27)	15.234	0.004
	Every week	187(10.59)	207(14.2)	483(11.08)		
	Every day	1548(87.66)	1227(84.16)	3776(86.65)		
Daily diet						
Carbonated drinks	1–3 times per week	34(1.94)	26(1.8) ^*#^	88(2.03)	32.552	< 0.001
	4–6 times per week	60(3.43)	46(3.18)	106(2.45)		
	4–6 times per week	12(0.69)	14(0.97)	34(0.79)		
	Every day	1645(93.95)	1361(94.06)	4099(94.73)		
Meat	1–3 times per week	319(18.2)	343(23.67) ^*#^	963(22.24)	32.58	< 0.001
	4–6 times per week	852(48.6)	593(40.92)	1851(42.74)		
	4–6 times per week	26(1.48)	39(2.69)	87(2.01)		
	Every day	556(31.72)	474(32.71)	1430(33.02)		
Tea consumption	Never/seldom	923(51.77)	673(45.44) ^*#^	2122(48.09)	38.511	< 0.001
	Drink monthly	127(7.12)	105(7.09)	288(6.53)		
	Drink weekly	497(27.87)	550(37.14)	1408(31.91)		
	Drink occasionally on special occasions	236(13.24)	153(10.33)	595(13.48)		
Respiratory exposure						
Heating or cooking fuel exposure	Both	773(43.23)	712(48.11) ^*#^	1896(42.97)	13.66	0.008
	Coal	896(50.11)	674(45.54)	2242(50.82)		
	Wood/Charcoal	119(6.66)	94(6.35)	274(6.21)		
Secondhand smoke exposure	Every day	53(2.97)	31(2.1) ^*#^	110(2.49)	24.254	< 0.001
	Never	473(26.47)	293(19.82)	1056(23.95)		
	1–5 times per week	1261(70.57)	1154(78.08)	3244(73.56)		
Smoking	No	1595(91.3)	1306(90.01)	3917(90.73)	1.575	0.455
	Yes	152(8.7)	145(9.99)	400(9.27)		

Note: Compared with Normal, *P < 0.05. Compared with Osteopenia, #*P* < 0.05

### Differences in body composition of age subgroups

Because being underweight is a known factor affecting osteoporosis, only those with a BMI greater than 18.5 Kg/m^2^ were included in the analysis of body composition. We found differences in body composition among three groups: normal, osteoporosis, and osteopenia. Further analysis of the body composition indicators of the groups aged < 45 and ≥ 45 revealed differences in body fat percentage, non-fat mass, muscle mass fraction, muscle mass, obesity level, VFI, body water percentage, and basal metabolism in both males and females (supplementary Table S1). However, in the population under the age of 45, there were not many differences in the various body composition indicators (such as body fat percentage, non-fat mass, muscle mass, obesity level, and VFI), except that visceral fat showed variations between osteoporosis and normal group in females (Table 2).

**Table 2:** Analysis of differences in body composition indicators for participants (*n* = 1717) with BMI > 18.5 and age < 45

Body composition	Male (*n* = 520)					Female (*n* = 1197)				
	Normal (*n* = 160)	Osteoporosis (*n* = 66)	Osteopenia (*n* = 294)	F	*P*	Normal (*n* = 438)	Osteoporosis (*n* = 93)	Osteopenia (*n* = 666)	F	*P*
Body weight	69.65 ± 10.54	73.78 ± 13.94^*#^	67.39 ± 10.7	9.430	< 0.001	61.92 ± 10.9	62.90 ± 11.77^*#^	59.96 ± 10.60	6.04	0.003
BMI	25.74 ± 3.63	27.07 ± 4.24^*#^	24.82 ± 3.36	11.741	< 0.001	25.94 ± 4.21	26.67 ± 4.84^*#^	25.39 ± 4.11	5.033	0.007
Waist circumference	92.36 ± 11.07	94.68 ± 15.89	90.89 ± 10.36	3.236	0.04	90.03 ± 11.22	92.31 ± 13.13^*#^	89.34 ± 11.08	2.925	0.054
Body fat percentage	25.35 ± 6.25	27.1 ± 5.37^*#^	24.06 ± 5.97	7.776	0.001	37.64 ± 6.7	38.76 ± 8.52^#^	36.87 ± 7.14	3.709	0.025
Non-fat mass	51.63 ± 6.39	53.28 ± 7.81^*#^	50.79 ± 6.31	4.08	0.018	38.04 ± 4.18	37.79 ± 4.73	37.32 ± 4.52^*^	3.580	0.028
Muscle mass fraction	14.76 ± 3.88	15.94 ± 4.68^#^	13.77 ± 3.77	9.463	< 0.001	14.13 ± 3.71	14.20 ± 3.89	13.67 ± 3.71	2.358	0.095
Muscle mass	48.93 ± 6.09	50.51 ± 7.42^*#^	48.14 ± 6	4.088	0.017	35.87 ± 3.82	35.66 ± 4.39	35.23 ± 4.16^*^	3.437	0.033
Obesity level	17 ± 16.95	23.73 ± 19.63^*#^	12.87 ± 15.25	12.766	< 0.001	18.02 ± 19.13	21.11 ± 22.16^*#^	15.63 ± 18.77	4.443	0.012
VFI	10.55 ± 3.52	12.77 ± 3.35^*^	11.24 ± 3.50	11.266	< 0.001	6.68 ± 2.43	7.24 ± 2.69^*^	6.95 ± 2.40	3.109	0.045
Body water percentage	51.87 ± 3.96	50.55 ± 4.01^#^	52.13 ± 4.66	3.499	0.031	46.95 ± 3.7	46.24 ± 5.35	47.23 ± 3.74	2.852	0.058
Basal metabolism	1444.28 ± 180.52	1493.7 ± 239.37^#^	1413.68 ± 182.48	5.17	0.006	1163.03 ± 141.96	1161.03 ± 150.91^#^	1136.03 ± 143.27^*^	5.112	0.006

Note: BMI: body mass index; VFI: visceral fat index

Compared with Normal, *P < 0.05. Compared with Osteopenia, #*P* < 0.05

### Multivariate logistics regression analysis

**Fig. 2:**
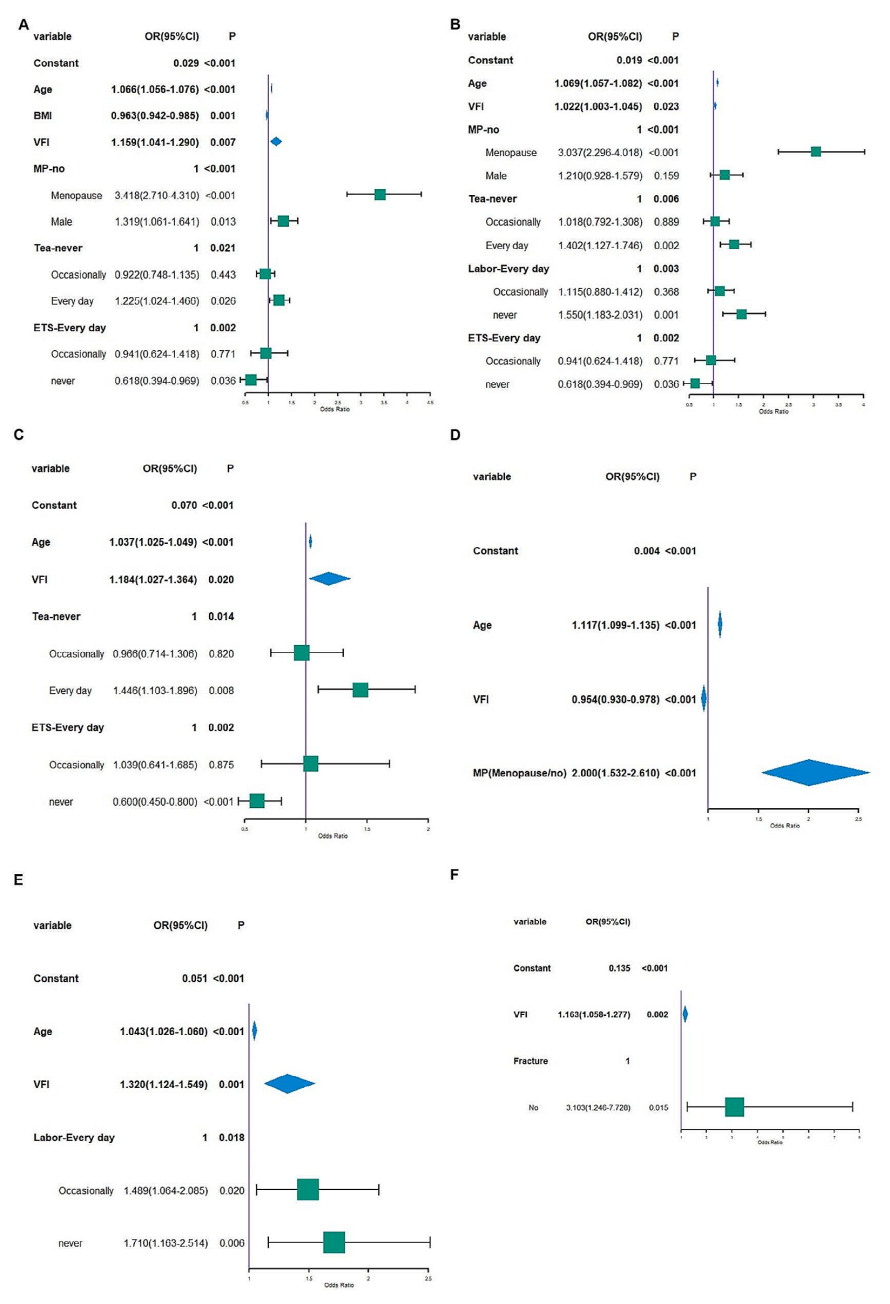
Factors affecting osteoporosis. VFI, visceral fat index; ETS, environmental tobacco smoke; MP, menopause. The osteoporosis was considered event y = 1. When analyzing the entire population, males were included as the third variable in the equation. (A) Factors affecting osteoporosis in the entire population of this survey. (**B**) Factors affecting osteoporosis in participants with BMI > 18.5 kg/m^2^. (**C**) Factors affecting osteoporosis in males with BMI > 18.5 kg/m^2^. (**D**) Factors affecting osteoporosis in females with BMI > 18.5 kg/m^2^. (**E**) Factors affecting osteoporosis in males with BMI > 18.5 kg/m^2^ and under 45 years of age. (**F**) Factors affecting osteoporosis in females with BMI > 18.5 kg/m^2^ and under 45 years of age

Multivariate logistics regression analysis found that age, BMI, VFI, menopause, tea, and environmental tobacco smoke (ETS) were influential factors for osteoporosis in the overall population, as shown in Fig. 2A. After stratifying by BMI > 18.5, BMI was no longer a significant factor. Instead, age, VFI, menopause, tea, ETS, and Labor were identified as influential factors for osteoporosis in those with BMI > 18.5 (Fig. 2B). By further dividing the population by gender and BMI > 18.5, the regression analysis showed that age, VFI, tea, and ETS were influential factors for osteoporosis in men (Fig. 2C), while age, VFI, and menopause were influential factors for women (Fig. 2D). After stratifying by age, the analysis found that VFI, Labor, and fracture were influential factors for men with BMI > 18.5 and age < 45 years old (Fig. 2E), while VFI and fracture were influential factors for women with BMI > 18.5 and age < 45 years old (Fig. 2F).

### Structural equation model

Owing to the diverse influential factors and the hazy correlation among the variables, we established the structural equation model to further explore the influential factors. In the theoretical model, we initially categorized the influential factors into five aspects, including general situations, body fat, daily diet, exercise, calcium supplementation, and respiratory exposure by conducting single-factor and multiple-factor analyses of osteoporosis. Additionally, exploratory factor analysis revealed that after dimension reduction, a total of 13 relevant factors were obtained, and the matrix of the relevant factors is shown in Table 3.

**Table 3:** The matrix of the relevant factors

	Heating	Second-hand smoke	Menopause	Age	Labor	Calcium sources and vitamin D	Dairy products	Osteoporosis	BMI	VFI	Fruits and vegetables	Meat	Tea consumption
Heating	-0.005												
Second-hand smoke	-0.156	0.022											
Menopause	0.428	0.219	-0.076										
Age	-0.401	-5.084	1.805	0.033									
Labor	-0.102	1.744	0.594	1.533	0.000								
Calcium sources and vitamin D	0.844	5.288	0.091	2.791	0.180	0.000							
Dairy products	-2.919	-4.960	1.380	-0.276	-0.096	0.176	0.000						
Osteoporosis	1.904	1.923	-0.951	-0.048	-1.129	1.249	-3.288	0.038					
BMI	-0.601	0.782	-1.608	-1.113	1.621	5.344	-1.353	0.176	0.000				
VFI	-0.303	1.135	-1.549	-0.679	1.499	5.337	-1.625	-0.520	0.000	0.000			
Fruits and vegetables	-0.761	-0.846	1.199	1.077	-0.623	0.130	-0.847	2.004	0.973	1.440	0.004		
Meat	0.463	2.824	0.295	0.175	0.349	1.681	-1.626	1.730	1.897	2.201	2.873	0.002	
Tea consumption	-0.265	2.358	-4.062	3.900	0.514	4.484	-4.534	3.473	3.809	4.067	4.168	-1.050	0.002

Note: BMI: body mass index; VFI: visceral fat index

**Fig. 3:**
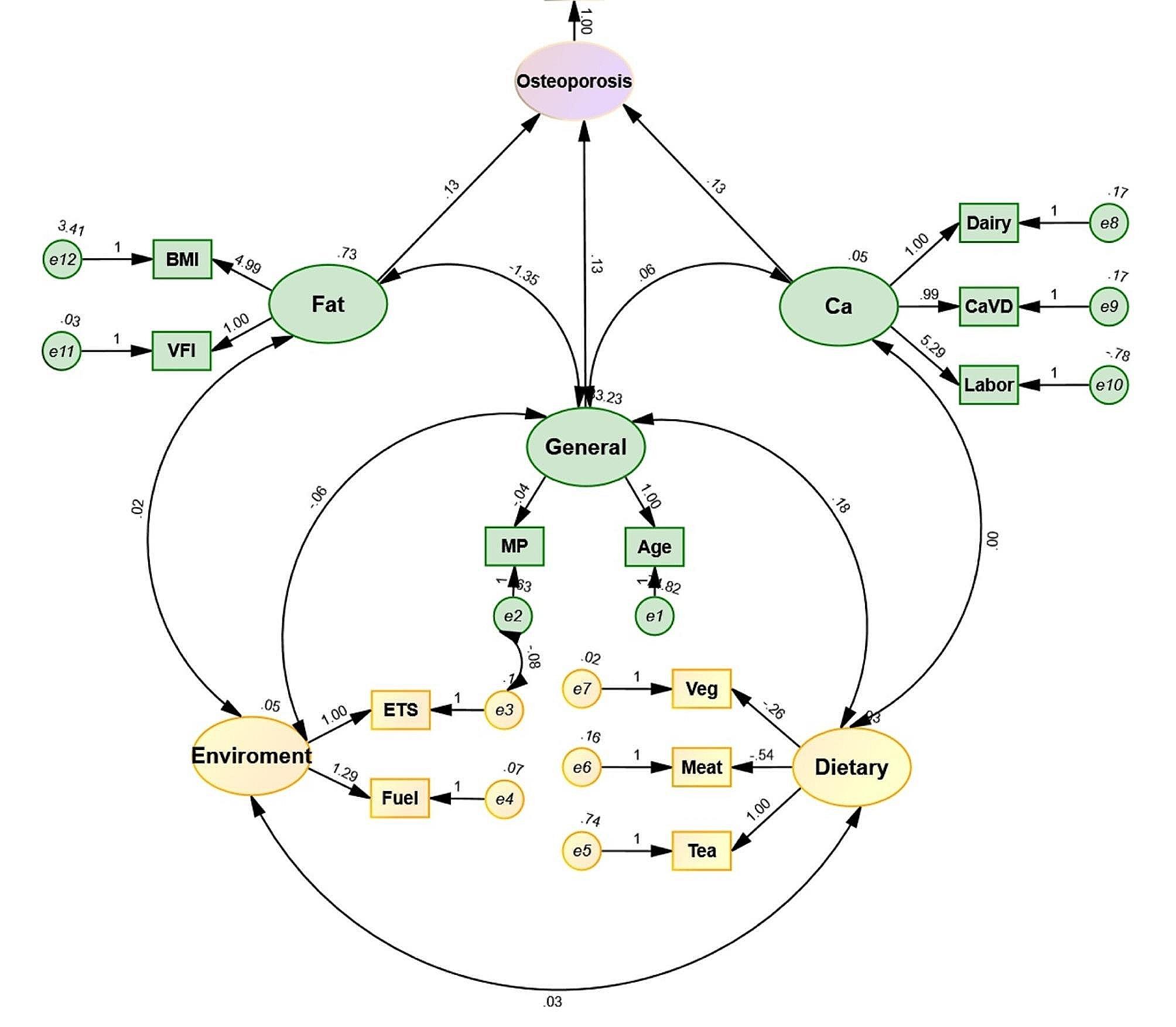
Structural equation model of factors affecting osteoporosis. VFI: visceral fat index; ETS: environmental tobacco smoke; MP: menopause. The osteoporosis was considered event y = 1. When analyzing the entire population, males were included as the third variable in the equation

In confirmatory factor analysis, there were 13 exogenous measurement variables (p) and 5 measurement variables (q), with a total of 171 data points and 55 free parameters (t). As t was less than the number of data points, the model was identifiable. The fit indices were as follows: RMSEA = 0.044, NFI = 0.973, RFI = 0.962, IFI = 0.977, TLI = 0.967, and CFI = 0.977, all of which were > 0.9, indicating a good model fit. The standardized path coefficients and factor loadings are presented in Fig. 3, and the regression weights of each factor are shown in Table 4, with R^2^ values greater than 0.5 for all indices except for tea and meat consumption. The structural equation model revealed that the general condition, body fat, and calcium sources and exercise directly affected osteoporosis, while respiratory exposure and daily diet indirectly affected osteoporosis through their mutual influence with the general condition, body fat, and calcium sources and exercise. The standardized path coefficient of the general condition (i.e., degree of influence) was the highest, followed by body composition, and finally, calcium sources and exercise.

**Table 4:** Regression weights among structural equation factors

Indicators		Estimate	S.E.	C.*R*.	*P*
Osteoporosis	General condition	0.128	0.013	9.693	< 0.001
	Body fat	0.13	0.035	3.717	< 0.001
	Calcium sources and exercise	0.128	0.035	3.683	< 0.001
Daily diet	Tea consumption	1			
	Meat	-0.542	0.111	-4.891	< 0.001
	Fruits and vegetables	-0.264	0.05	-5.277	< 0.001
Body fat	VFI	1			
	BMI	4.994	0.227	21.97	< 0.001
Calcium sources and exercise	Dairy product	1			
	Calcium sources and vitamin D	0.989	0.029	33.643	< 0.001
	Labor	5.293	0.351	15.064	< 0.001
General condition	Age	1			
	Menopause	-0.043	0.003	-12.406	< 0.001
Respiratory exposure	Second-hand smoke	*1*			
	Heating	1.293	0.168	7.718	< 0.001

Note: BMI: body mass index; VFI: visceral fat index

## Discussion

This study utilized various methods to explore the influencing factors of osteoporosis. Some of our results, such as the influencing factors of age, gender, menopause, education level, fractures, calcium or vitamin D supplements, labor, and dairy product intake, were consistent with previous findings [22, 23], while some results were revealed for the first time. Specifically, we found that body composition, especially visceral fat, exhibited an impact on osteoporosis, as shown through single-factor analysis, logistic regression analysis, and the structural equation model. The underlying mechanism deserves further exploration. It has been reported that in addition to body weight, the body composition and weight distribution of the trunk and legs have significant effects in preventing osteoporosis [22].

Osteoporosis is a disease commonly associated with aging. Menopause in women has long been the focus of research on this disease, as a lack of estrogen is linked to bone loss [24]. It is generally believed that adipocytes can produce sex steroids including estrogen, which can promote bone differentiation, inhibit the formation of osteoclasts, and induce apoptosis of osteoclasts [25]. However, this effect requires that the adipose tissue be subcutaneous, as estrogens in subcutaneous adipose tissue are associated with higher levels of circulating estrogens that have a positive effect on bone mass and mineralization [26]. Conversely, this effect is not observed in obese patients with high visceral fat, and instead, an increase in visceral adipose tissue is associated with a decrease in BMD [27]. Men with higher body weight due to the action of testosterone are subject to the same phenomenon [28, 29]. Furthermore, obese men tend to have lower levels of sex hormone-binding globulin, which increases levels of free sex steroids and has been associated with lower BMD [30]. Besides hormonal factors, inflammatory mechanisms also have a significant impact on the relationship between fat and osteoporosis [31]. Abdominal visceral adipose tissue contains higher levels of adipokines [32], which increase the number of inflammatory factors and lead to elevated bone resorption and decreased bone formation [33]. This accelerates the progression of osteoporosis [34]. Therefore, reducing the accumulation of visceral fat, maintaining a proper weight and fat distribution, consuming a balanced diet, and increasing physical activity can help prevent and delay the occurrence of osteoporosis [1].

This study found that previous chronic disease would affect osteoporosis, which is consistent with previous studies on diabetes [35], elevated thyroid hormone levels [36], chronic gastrointestinal diseases [37], etc. These previous diseases can increase the risk of osteoporosis. However, in addition to the related mechanisms of comorbidities, obesity also has a significant impact on these diseases. Further research is essential to determine whether obesity has a causal role in the development of osteoporosis through its complex pathway. Smoking might elevate the risk of osteoporosis and fractures. Smoke exposure could potentially increase the level of superoxide free radicals and decrease the activity of intracellular glutathione reductase in mesenchymal stem cells, which could hinder osteogenic differentiation [38]. Moreover, it has been demonstrated that smoking-related inflammation could induce changes in the expression of genes related to bone remodeling [39]. However, this study did not find a relationship between osteoporosis and smoking or alcohol consumption, possibly because the population in this area influenced by religious beliefs is unwilling to disclose the true situation to investigators. However, the study found that exposure to inhaled substances in the environment, such as burning fuels such as firewood/charcoal and the way of winter heating, is related to osteoporosis, which may be similar to the principle of smoking.

There are some limitations to this study. For example, this epidemiological survey was conducted in rural areas and the BMD was measured using a portable bone density meter. The diagnosis of osteoporosis was made based on the BMD results rather than the gold standard diagnosis method. Although the results are reliable, there may be deviations in defining population diseases. Further studies are warranted.

## Conclusions

We demonstrated that age, female menopause, and obesity were influencing factors of osteoporosis. Particularly, VFI has a significant impact on osteoporosis. Our findings may provide experimental evidence for early prevention and treatment of osteoporosis.

### Electronic supplementary material

Below is the link to the electronic supplementary material.

Supplementary Material 1

## Data Availability

The datasets generated during and/or analysed during the current study are available from the corresponding author on reasonable request.

## Abbreviations

BMI: body mass index
BMD: bone mineral density
VFI: visceral fat index
