# Supplementary material for: Structural equation model analysis of the effect of visceral fat on osteoporosis
Source: J Orthop Surg Res. 2024 Jul 16;19:408. doi: 10.1186/s13018-024-04888-5 (PMC11253341; doi:10.1186/s13018-024-04888-5)
Supplement: Supplementary file 1 — Supplementary Material 1 [file 13018_2024_4888_MOESM1_ESM.docx]

**Supplementary Table S1. Analysis of differences in body composition indicators for participants (n=5757) with BMI>18.5 and age≥45.**

| Body composition | Male (n=2342) | | | | | Female (n=3415) | | | | |
| --- | --- | --- | --- | --- | --- | --- | --- | --- | --- | --- |
|  | Normal (n=567) | Osteoporosis (n=446) | Osteopenia (n=1329) | *F* | *P* | Normal (n=608) | Osteoporosis (n=803) | Osteopenia (n=2004) | *F* | *P* |
| Body weight | 67.68±11.86 | 68.24±13.86 | 65.6±11.38 | 11.081 | <0.001 | 58.04±12.54 | 64.52±11.15^*#^ | 59.83±10.93 | 59.465 | <0.001 |
| BMI | 25.6±3.84 | 25.91±4.43^#^ | 24.93±3.7 | 13.235 | <0.001 | 25.45±4.98 | 26.95±4.31^*#^ | 25.62±4.29 | 24.393 | <0.001 |
| Waist circumference | 93.51±11.27 | 95.47±13.05^*#^ | 92.5±10.76 | 11.549 | <0.001 | 94.7±11.56 | 96.07±11.67^*#^ | 94.09±11.38 | 6.929 | 0.001 |
| Body fat percentage | 25.22±5.83 | 26.42±6.28^*#^ | 24.89±6.18 | 10.41 | <0.001 | 37.60±8.14 | 38.93±7.42^*#^ | 37.65±7.57 | 7.08 | 0.001 |
| Non-fat mass | 50.14±6.57 | 49.65±7.71^#^ | 48.86±6.69 | 7.646 | 0.001 | 35.49±5.11 | 38.81±4.78^*#^ | 36.75±4.89 | 79.34 | <0.001 |
| Muscle mass fraction | 14±3.95 | 13.99±4.57^#^ | 13.3±3.96 | 8.252 | <0.001 | 12.87±4.47 | 14.68±3.77^*#^ | 13.25±3.9 | 39.286 | <0.001 |
| Muscle mass | 47.52±6.25 | 47.05±7.33^#^ | 46.3±6.36 | 7.63 | 0.001 | 33.55±4.68 | 36.59±4.39^*#^ | 34.7±4.49 | 78.921 | <0.001 |
| Obesity level | 16.08±17.16 | 17.83±20.38^*#^ | 13.45±17.04 | 11.734 | <0.001 | 15.7±22.46 | 22.23±19.57^*#^ | 16.49±19.5 | 22.165 | <0.001 |
| VFI | 13.75±3.54 | 14.31±3.84^*#^ | 13.34±3.68 | 11.869 | <0.001 | 7.84±2.75 | 8.3±2.54^*#^ | 7.75±2.53 | 10.441 | <0.001 |
| Body water percentage | 51.12±4.38 | 49.33±4.79^*#^ | 50.8±4.89 | 20.507 | <0.001 | 45.81±4.9 | 45.96±4.13 | 46.14±4.48 | 1.539 | 0.215 |
| Basal metabolism | 1374.79±198.25 | 1363.71±235.55^#^ | 1336.21±197.72 | 8.091 | <0.001 | 1063.35±167.98 | 1175.55±152.95^*#^ | 1103.12±153.57 | 89.634 | <0.001 |

Note: BMI, body mass index; VFI, visceral fat index.
